# Prevalence and Distribution of Oral Mucosal Lesions by Sex and Age Categories: A Retrospective Study of Patients Attending Lebanese School of Dentistry

**DOI:** 10.1155/2018/4030134

**Published:** 2018-05-17

**Authors:** Sami El Toum, Antoine Cassia, Nermine Bouchi, Issam Kassab

**Affiliations:** ^1^Department of Oral Pathology and Diagnosis, Lebanese University, Beirut, Lebanon; ^2^Faculty of Pharmacy, Lebanese University, Beirut, Lebanon

## Abstract

**Background:**

Prevalence and distribution of oral mucosal lesions in a sample of Lebanese population attending the School of Dentistry of Lebanese University is necessary to evaluate their oral health situation.

**Objectives:**

The aim of the present study was to determine the prevalence and distribution of oral mucosal lesions of patients attending the School of Dentistry.

**Methods:**

A descriptive study was carried out by retrospectively examining a total of 231 medical and clinical examination record files of patients, attending the School of Dentistry Lebanese University for multidisciplinary dental treatments. 178 medical records were retained. Each medical and clinical examination record was done by an undergraduate student and then evaluated by a doctor. The record file included a civil status, chief complaint, medical history, and extraoral and intraoral clinical examination during the period between October 2014 and May 2015. Exclusion criteria were lack of written information in their medical and clinical examination record and being nonevaluated by a doctor. Data regarding age, gender, socioeconomic status, chief complaint, systemic diseases, and drugs intake were collected by using a questionnaire while the type of extraoral and oral mucosal lesions by clinical examination.

**Results:**

The sample consisted of 102 (57.3%) females and 76 (42.7%) males. The age ranged from 10 to 92 years with a mean age of 40.1 years. Among these subjects, 110 (61.8%) presented with one or more lesions. All patients were Lebanese. The most common lesion diagnosed was coated/hairy tongue affecting 17.4% of the subjects, followed by melanotic macule (11.2%), gingivitis (9.6), linea alba (6.2%), tongue depapillation (5.1), leukoplakia (5.1), traumatic fibroma (4.5), frictional keratosis (3.9%), fissured tongue (3.9%), hemangiomas (3.9%), Fordyce granules (3.9%), dry mucosa (3.4), angular cheilitis (2.2), gingival hyperplasia (2.2), and crenulated tongue (1.7%). Overall, the prevalence of oral mucosal lesions did not significantly differ between sex and age groups.

**Conclusions:**

The high prevalence of oral mucosal lesions necessitates adequate awareness and management of these lesions in the general population. Dental clinicians should be knowledgeable and familiar with the etiopathogenesis, clinical presentation, diagnosis, and management of these lesions.

## 1. Introduction

Oral mucosal lesion (OML) is known as any abnormal alteration in color, surface aspect, swelling, or loss of integrity of the oral mucosal surface. Although a proportion of OMLs are benign and require no active treatment, some may present with significant pathology. Of particular importance are oral potentially malignant disorders which may progress into malignancy. Besides, OMLs can interfere with daily quality of life in affected patients through impacts on mastication, swallowing, and speech with symptoms of burning, irritation, and pain [1]. OMLs have many etiologies as bacterial or viral or fungal infections, local trauma or irritation, systemic diseases, and excessive consumption of tobacco, betel quid, and alcohol [2, 3].

In literature, epidemiological studies of OMLs are still few when compared with reports regarding dental caries or periodontal diseases [4]. The prevalence of OMLs in general population globally varies significantly across different countries and areas, ranging from 4.9% to 64.7% [1–3, 5–7]. Feng et al. [1] found that the overall prevalence of OMLs in a Chinese population was 10.8%. Amarodi et al. [5] reported a prevalence of 31.7% of OMLs in the teenager group.

This gap is even more apparent in case of children and adolescents, where studies focus above all on cancer patients or samples with specific chronic diseases. Also, there is a tendency of using different experimental methods, nonstandardized diagnostic criteria, and small samples which lead to a controversial and underestimated prevalence of OMLs in adolescents. However, the literature demonstrates that the prevalence of OMLs seems to change and increase with age along with the development of bad habits [5].

The investigation of OML prevalence in specific population groups is mandatory to understand its extension and characteristics, but it is also essential for the improvement of oral health promotion and prevention programs for specific age groups, as recommended by the World Health Organization (WHO) [5, 8].

Epidemiologic studies provide information that is important to understand the prevalence, incidence, and severity of oral disease in a specific population, but the results of such studies have rarely been published worldwide and a wide difference in the results [9].

To the best of our knowledge, there have been no broad population-based epidemiological studies about the prevalence of OMLs in Lebanese population that have not been selected for age, gender, or risk habits. Thus, the objectives of this study were to investigate the prevalence and distribution of OMLs in a Lebanese sample attending the School of Dentistry to seek a dental treatment. This study helps to elaborate the adequate management of the prevention protocol and the needed treatment for this population.

## 2. Materials and Methods

We retrospectively analyzed a total of 231 medical and clinical examination records of patients attending the Lebanese University for multidisciplinary dental treatments. The informed consent was obtained from each participant, after recalling him or her by phone and explaining the objective of this study, to permit the use of the data registered in his or her file.

The medical and clinical examination records were filled by an undergraduate student and then evaluated by a doctor of the Department of Oral Pathology and Diagnosis. Doctors of the department had undergone the same training and therefore permitted the standardization of the procedures of medical observation and clinical examination. The calibration was done after multiple sessions of illustrating and interpreting different lesions to perform an identical teaching method and description. Each patient's examination was conducted under artificial lighting on a dental chair and with a mirror. OMLs were classified following the WHO criteria [8, 10]. Exclusion criteria were lack of written information in the medical and clinical examination records or not evaluated by a doctor of the department. From 231 medical records selected from October 2014 until May 2015, fifty-three files were ruled out.

Each medical and clinical examination record file reports the patient's civil status, chief complaint, medical observation, drugs intake, and extraoral and intraoral clinical examination.

The civil status consists of age, gender, present and previous occupation, and address. Chief complaint with its anamnesis that let the individual consults the school of dentistry was performed. The medical history and the previous surgery and hospitalized period were collected by questioning the patient. Extraoral examination of the facial disharmony, cutaneous, temporomandibular articulation, and palpation of the lymph nodes was done. Also, intraoral examination noted the presence of any lesions or an anatomical variation on the oral mucosa. Clinical examinations were performed according to the WHO guideline [8].

The elements to evaluate during the questionnaire included general status, age, gender, systemic diseases, drugs used, and prosthetic or other appliances used. During the clinical examination, the following elements were noted: features of the lesion, anatomical site, extension, etiological or related factors, dental status, trauma, use of prosthesis, and whether or not these were well adapted. All oral lesions were treated in the Department of Oral Pathology and Diagnosis if treatment is requested.

All the obtained data were analyzed statistically by using a Statistical Package for Social Sciences version 20 (SPSS Inc. Chicago, IL, USA).

A descriptive analysis of the sample was first performed using means (±standard deviation (SD)) for continuous variables and frequencies (proportions) for categorical variables. Different intraoral and extraoral findings on clinical examination were reported.

The chi-square test and Fisher's exact test were used to compare proportions of OMLs between males and females and between age groups (age being categorized into three groups: 30 years or less, between 30 and 60 years, and more than 60 years). A *p* value less than 0.05 was deemed statistically significant.

## 3. Results

A total of 178 patients were recruited into the study, among whom 76 (42.7%) were male. They were aged between 10 and 92 years with a mean age of 40.1 years (±17.7) (Table 1). The analysis shows that 44.9% of patients consulting the School of Dentistry were coming from the closest region around the University, and 43.6% were employed with a middle-to-low socioeconomic status.

The chief complaint of 24.1% of patients was a tooth or gingival pain followed by restorative and cosmetic dentistry (13.3% each), to specialized consultation for mastication problems or mucosal lesions (7.8%), only 2.4% consulted for a checkup as illustrated in Table 2.

The patients' medical history revealed the presence of hypertension among 7.3% of the cases, allergies in 7.9% (drug allergies such as aspirin and penicillin, seasonal allergies, and food allergies), diabetes in 5.6%, asthma in 3.9%, cardiac diseases in 3.4%, and dyslipidemia in 2.2% of the cases. Furthermore, 47.2% of the patients suffered different other systemic disorders such as thyroid problems, gastrointestinal problems, cancer, osteoporosis, and surgery histories (Table 3).

Many of them were on chronic cardiovascular medications and other drugs as shown in Table 3. Drugs intake were mainly for antihypertensives (10.7%), antithrombotics (8.4%), vitamins (7.9%), antacids (5.6%), analgesics (5.1%), antidiabetics (4.5%), and antihypercholesterolemic (3.4%) (Table 3).

The extraoral examination revealed lentigo and nevus in most patients, lymph nodes, and varicella or traumatic scars (Table 4).

Following the intraoral examination, different pathologies were noted (Table 4). The most common lesion was coated/hairy tongue affecting 17.4% of the subjects, followed by melanotic macule (11.2%), gingivitis (9.6%), linea alba (6.2%), tongue depapillation (5.1), leukoplakia (5.1), traumatic fibroma (4.5), frictional keratosis (3.9%), fissured tongue (3.9%), hemangiomas (3.9%), Fordyce granules (3.9%), dry mucosa (3.4%), angular cheilitis (2.2), gingival hyperplasia (2.2), aphthous (1.7%), crenulated tongue (1.7%), leukoedema (1.1%), ankyloglossia (1.1%), fistula (1.1), prosthetic stomatitis (1.1%), mandibular tori (1.1), mucoceles (1.1%), and lingual varices (0.6%).

In the majority of the cases, only one lesion was found (42%); however, some patients exhibited more than one oral lesion simultaneously.

As shown in Table 5, the prevalence of extraoral and intraoral lesions did not significantly differ between males and females except for melanotic macules which were more frequent among males (17.1%) than females (6.9%) (*p*=0.032).

Intraoral lesions were more frequent among those aged more than 60 years (73.1%) than those aged between 30 and 60 years (61.4%) or less than 30 years (58.1%), but the analysis did not show a significant statistical difference (*p*=0.412). Gingivitis was more prevalent among younger groups (16.1% for those ≤30 years versus 6.8% for those between 30 and 60 years and 0% for those >60 years; *p*=0.032), whereas tongue depapillation was more prevalent among the elderly (15.4%) than those ≤30 years (4.8%) (*p*=0.055). More details are shown in Table 6.

The prevalence of extraoral findings was not significantly different between age groups (Table 6) except for cicatrix, which was more frequent among younger groups (14.5%) than older ones (3.4% and 3.8%) (*p*=0.032).

## 4. Discussion

The prevalence of OMLs in the Lebanese sample was 61.8%. Epidemiologic studies have demonstrated a wide variety in prevalence rates in oral lesions in different populations due to various habits. It has been reported that OMLs may affect 4.9% to 64.7% of individuals having various habits, depending on the population studied [9, 11–13]. Andreason [14] found a prevalence of OMLs of 9.9%. Patil et al. [11] showed that 64% of patients presented one or more oral lesions. These lesions can be associated with tobacco, betel nut consumption, or secondary to trauma and prosthesis [7, 11, 15]. In our study, the population was patient attending the School of Dentistry-Lebanese University for multidisciplinary dental treatment with a middle-to-low socioeconomic status. These factors can increase the prevalence of OMLs in comparison to the general Lebanese population.

The overall prevalence of OMLs was found to be higher in older individuals than younger individuals, and it can be related to different habits acquired with age. Chewing, smoking, and consumption of alcoholic beverages have become a common social habit in India [16]. Pratik and Desai [16] found that the prevalence of habits in Indian population was 51.4% including both the sexes, and the prevalence of OMLs was 9.9%. The oral lesions were more frequently observed between 65 and 70 years [7, 11, 17]. The mean age of a large portion of OMLs, such as fissured tongue, lingual papillitis, candidiasis, lichen planus, melanin pigmentation, and burning mouth syndrome, was over 60 years old [18]. In our population, 50.0% of the participants have an age between 30 and 60 years and only 14.8% more than 60 years our prevalence was 61.8% and can be related to the high percentage of old patients.

Patil et al. [11] showed males were more affected than females, and this difference was clinically not significant (*p* > 0.05). In our study, oral lesions affected 64.5% of males and only 59.8% of females, but the difference was not statistically significant.

To our knowledge, it is the first study on a Lebanese sample which showed epidemiologic figures on OMLs prevalence and distribution. Our population was patients who consult the Dental school of the Lebanese University, with a different chief complaint, for a dental treatment which probably increases the prevalence of OMLs.

Patil et al. in their study about a geriatric sample of Indian population revealed 64% of the patients presented with one or more oral lesions, associated with tobacco, betel nut consumption, and lesions secondary to trauma and prosthesis [9]. Also in our population, 61.8% presented with one or more oral lesions, and 50% were smokers.

In this study, the most common lesions were coated tongue with 17.4% and melanotic macule (11.2%), gingivitis (9.6%), linea alba (6.2%), tongue depapillation (5.1), smoking keratosis (5.1), traumatic fibroma (4.5), frictional keratosis (3.9%), fissured tongue (3.9%), hemangiomas (3.9%), and Fordyce granules (3.9%). Tortorici et al. in their study on Caucasian population [4] found coated/hairy tongue in 16.7% of the subjects, lingual varices (16.3%), secondary herpes lesions (8.1%), aphthous ulcers (7.9%), Fordyce granules (7.2%), frictional keratosis (5%), candidiasis (4.9%), fibroepithelial hyperplasia (4.6%), squamous papilloma (3.8%), traumatic ulcers (3.7%), leukoplakia (3.2%), fissured tongue (3.2%), hemangiomas (2.7%), and morsicatio buccarum (2.5%). In geriatric Indian [9] and Thailand [7] population, the most common OMLs observed were smoker's palate (43%), denture stomatitis (34%), oral submucous fibrosis (30%), frictional keratosis (23%), leukoplakia (22%), and pyogenic granuloma (22%). Hard palate was the most commonly affected site (23.1%).

Feng et al. [1] found the most common type of OMLs was fissured tongue (prevalence of 3.15%), followed by recurrent aphthous (1.48%), traumatic ulcer (1.13%), and angular cheilitis (0.86%). The two most common potentially malignant disorders were oral lichen planus (0.81%) and leukoplakia (0.22%). In a teenaged group, Amadori et al. found the most frequent were aphthous ulcers (18%), traumatic ulcerations (14.3%), herpes simplex virus (11%), geographic tongue (9.6%), candidiasis (5.5%), and morsicatio buccarum (4.7%). Papilloma virus lesions (1.7%), piercing-related lesions (4%), multiforme erythema (0.13%), oral lichen planus (0.13%), and granular cell tumour (0.06%) were also diagnosed [5].

The prevalence of each OML varied from study to other as shown above because with age many alterations of the oral mucosal lesion can be induced. Also, the selection of the population can affect the prevalence of oral lesions. To compare two populations, they must have the same percentage of habits, mean age, and the same distribution of individuals following the age group.

Concerning the occurrence and distribution of systemic diseases, a study in Brazil showed that 24.6% participants were hypertensive, 15% hypercholesterolemic, 5.6% diabetic, and 4.5% positive for hepatitis. The prevalence of drugs intake was 22.2% for antihypertensive drug use, 6.7% diuretics, 3.9% hypoglycemic, 3.2% contraceptives, 3% analgesics, 1.6% nonsteroid anti-inflammatory drugs, and 0.4% antibiotics [18]. In our study, 7.3% were hypertensive, 2.2% hypercholesterolemic, 5.6% diabetic, and 14% allergic. The prevalence of drugs intake was 10.7% for antihypertensives, 3.9% antidiabetics, 5.1% analgesics, 3.4% NSAIDs, and 4.5% antibiotics. In the Lebanese population, analgesics, anti-inflammatories, and antibiotics intake was higher than in other studies.

In diabetic patients, a series of oral mucosa alterations have been reported, including periodontal and oral mucosal diseases that favor infections such as candidiasis, salivary gland dysfunction, altered taste, glossodynia, and stomatopyrosis [19]. The prevalence of OMLs in patients with diabetes mellitus such as lichen planus and recurrent aphthous ulceration has been of 80% in diabetic patients, although the actual prevalence is rarely addressed in clinical studies [19].

Our study is limited by its retrospective nature where information and classification biases might exist and results cannot be generalized to the general population.

## 5. Conclusion

The prevalence of oral mucosal lesions varied widely among populations and studies. Many factors can influence the result as the mean age of the sample. Older populations have relatively a higher percentage of oral mucosal lesions than would younger populations. Habits, like tobacco smoking, alcohol consumption, which further increase with age, can also increase the incidence of oral mucosal lesions. Systemic diseases and drugs can be related to oral mucosal lesions prevalence. Our study was the first epidemiologic study in Lebanon about the prevalence of oral mucosal lesions and their relation to many factors. Larger community-based studies should be conducted to estimate more representative percentages of oral lesions.

## Figures and Tables

**Table 1:** Total sample description (*n*=178).

Characteristic	Frequency (percentage)
Age at consultation (mean ± SD years; (range))	40.1 ± 17.7 (10–92)
≤30	62 (35.2)
(30–60)	88 (50.0)
>60	26 (14.8)

Gender	
Male	76 (42.7)
Female	102 (57.3)

Occupation	
Student	32 (21.5)
Employee/self-employed	65 (43.6)
Housewife/retired	52 (34.9)

Address (closest to farthest)	
Baabda Hadath	80 (44.9)
Beirut Khaldeh	60 (33.7)
Jabal Jounieh	19 (10.7)
Bekaa Akkar	16 (9.0)
Others	3 (1.7)
Tobacco smokers	89 (50.0)

**Table 2:** Patients' chief complaints (*n*=178).

Chief complaint	Number of subjects (percentage)
Pain: tooth or gingival	40 (24.1)
Others	34 (20.5)
Restauration prosthesis dentistry	22 (13.3)
Esthetics	22 (13.3)
Assistant prosthesis	21 (12.7)
Specialized consultation mastication	13 (7.8)
Periodontal problem	10 (6.0)
Checkup	4 (2.4)

**Table 3:** Patients' medical and medications history (*n*=178).

Characteristic	Frequency (percentage)
*Medical history*	
Allergy	14 (7.9)
Hypertension	13 (7.3)
Diabetes	10 (5.6)
Asthma	7 (3.9)
Cardiac disease	6 (3.4)
Dyslipidemia	4 (2.2)
Other diseases	84 (47.2)

*Medications*	
Antihypertensives	19 (10.7)
Antithrombotics	15 (8.4)
Vitamins	14 (7.9)
Antacids	10 (5.6)
Analgesics	9 (5.1)
Antidiabetics	8 (4.5)
Antihypercholesterolemic	6 (3.4)
Antiasthmatics	6 (3.4)
Anti-inflammatories	6 (3.4)
Antidepressants	5 (2.8)
Anxiolytics	4 (2.2)
Bisphosphonates	3 (1.7)

**Table 4:** Lesion findings on intraoral and extraoral examinations (*n*=178).

	Number of subjects (percentage)
*Oral mucosal lesions*	110 (61.8)
Coated tongue	31 (17.4)
Melanotic macule	20 (11.2)
Gingivitis	17 (9.6)
Linea alba	11 (6.2)
Tabagic keratosis	9 (5.1)
Tongue depapillation	9 (5.1)
Traumatic fibroma	8 (4.5)
Fordyce granules	7 (3.9)
Petechia, hemangioma	7 (3.9)
Fissure tongue	7 (3.9)
Frictional keratosis	7 (3.9)
Dry labial and mouth	6 (3.4)
Angular cheilitis	4 (2.2)
Gingival hyperplasia	4 (2.2)
Aphthous	3 (1.7)
Crenulated tongue	3 (1.7)
Leucoderma	2 (1.1)
Ankyloglossia	2 (1.1)
Fistula	2 (1.1)
Stomatitis under prosthesis	2 (1.1)
Mandibular tori	2 (1.1)
Mucocele	2 (1.1)
Impression of teeth on labial mucosa	1 (0.6)
Lingual varicosity	1 (0.6)

*Extraoral lesions*	82 (46.1)
Lentigo nevus	46 (25.8)
Cicatrix	13 (7.3)
Lymph nodes	10 (5.6)
Varicosity	7 (3.9)
Acne	7 (3.9)
Dry lips	6 (3.4)

**Table 5:** Most frequent extraoral and oral mucosal lesions by sex categories.

	Females (*n*=102)	Males (*n*=76)	*p* value
*Oral mucosal lesions*	61 (59.8)	49 (64.5)	0.526^†^
Coated tongue	16 (15.7)	15 (19.7)	0.481^†^
Melanotic macule	7 (6.9)	13 (17.1)	0.032^†^
Gingivitis	9 (8.8)	8 (10.5)	0.702^†^
Linea alba	7 (6.9)	4 (5.3)	0.457^‡^
Tabagic keratosis/leucoderma	5 (4.9)	4 (5.3)	0.587^‡^
Tongue depapillation	6 (5.9)	3 (3.9)	0.734^‡^

*Extraoral lesions*	50 (49.0)	32 (42.1)	0.360^†^
Lentigo naevus	31 (30.4)	15 (19.7)	0.108^†^
Cicatrix	7 (6.9)	6 (7.9)	0.794^†^
Lymph nodes	8 (7.8)	2 (2.6)	0.121^‡^
Varicosity	2 (2.0)	5 (6.6)	0.120^‡^
Acne	3 (2.9)	4 (5.3)	0.341^‡^
Dry lips	3 (2.9)	3 (3.9)	0.512^‡^

^†^Pearson chi-square test; ^‡^Fisher's exact test.

**Table 6:** Most frequent extraoral and intraoral lesions by age groups.

	Age groups			*p* value
	≤30 years (*n*=62)	30–60 years (*n*=88)	>60 years (*n*=26)	
*Oral mucosal lesions*	36 (58.1)	54 (61.4)	19 (73.1)	0.412^†^
Coated tongue	6 (9.7)	19 (21.6)	6 (23.1)	0.123^†^
Melanotic macule	8 (12.9)	12 (13.6)	0	0.140^†^
Gingivitis	10 (16.1)	6 (6.8)	0	0.032^†^
Linea alba	7 (11.3)	4 (4.5)	0	0.125^‡^
Tabagic keratosis/leucoderma	3 (4.8)	6 (6.8)	0	0.521^‡^
Tongue depapillation	3 (4.8)	2 (2.3)	4 (15.4)	0.055^‡^

*Extraoral lesions*	30 (48.4)	38 (43.2)	13 (50.0)	0.744^†^
Lentigo naevus	14 (22.6)	22 (25.0)	10 (38.5)	0.285^†^
Cicatrix	9 (14.5)	3 (3.4)	1 (3.8)	0.032^‡^
Lymph nodes	1 (1.6)	8 (9.1)	1 (3.8)	0.138^‡^
Varicosity	1 (1.6)	4 (4.5)	2 (7.7)	0.262^‡^
Acne	4 (6.5)	2 (2.3)	0	0.248^‡^
Dry lips	5 (8.1)	1 (1.1)	0	0.066^‡^

^†^Pearson chi-square test; ^‡^Fisher's exact test.
